# Toward Reliable Lesion Labeling Standards for AI in Nuclear Medicine

**DOI:** 10.2967/jnumed.125.271329

**Published:** 2026-05

**Authors:** Remco J. Poelarends, Jorn A. van Dalen, Riemer H.J.A. Slart, Brian N. Vendel, Walter Noordzij, Joris D. van Dijk

**TO THE EDITOR:** With keen interest, we read the special contribution entitled “Recommendations for Standardizing Nuclear Medicine Terminology and Data in the Era of Theranostics and Artificial Intelligence” by Bradshaw et al. (1). The authors present highly relevant recommendations as the unprecedented growth of the field, driven by advances in radiopharmaceutical therapy and the emergence of artificial intelligence (AI), depends on large, well-structured datasets. Data standardization supports multi-institutional sharing, reproducibility of results, and comparability of clinical outcomes.

While we fully support the proposed adoption of controlled vocabularies and standardized data frameworks, we argue that another pressing challenge lies in the lack of consistency and reliability in lesion-labeling approaches applied to medical imaging data. Accurate and consistent lesion labeling, referring to labels that are reproducible across readers and reflect the true nature of each lesion, is critical for both the development and validation of AI algorithms. In the development phase, poor label quality can unfairly penalize model performance (2). For example, when patterns are present in the data, incorrect labels can provide conflicting information to the algorithm. This would limit its ability to learn meaningful and generalizable features. In the evaluation phase, the use of unreliable labels can lead to uncertainty in the calculated performance metrics, making it difficult to assess the clinical value of an AI algorithm (3). We acknowledge that high-quality labeling requires substantial time and resources, yet this investment is crucial for reliable AI development. Notably, models that are trained on unreliable labels are not necessarily of poor quality; rather, the evaluation reflects the model’s performance on the dataset with that specific labeling.

To illustrate the challenge of lesion labeling, we built on the use case described by Bradshaw et al. regarding prostate-specific membrane antigen (PSMA) and conducted a global literature search on AI algorithms for lesion detection or classification using PSMA PET/CT. The search query used on Europe PubMed central is provided in the supplemental materials (available at http://jnm.snmjournals.org). We found 17 articles of which 14 reported on model development for lesion detection or segmentation and 3 focused on lesion classification or tracking lesions over multiple scans. Although dataset sizes have increased, thereby enabling better algorithm development, the quality of the labeling methodology does not follow this trend, as summarized in Supplemental Table 1. Most of the 17 found articles relied on a single reader per scan (*n* = 8) or did not clearly describe their methodology (*n* = 4), whereas only 4 articles used multireader consensus. Remarkably, just 1 article incorporated follow-up imaging for labeling, which was required by its lesion-tracking purpose. These findings underscore that systematic, transparent, and robust labeling strategies are still rarely implemented for PSMA PET algorithms.

The reliance on single-reader labeling raises important questions about labeling quality and reproducibility. One may argue that labels from a single reader do not necessarily imply low-quality labels. However, a higher number of readers, ideally combined with additional clinical and imaging data, tends to improve the label quality as demonstrated by, for example, breast imaging where double reading is standard of care in some countries to improve diagnostic reliability (4). Importantly, algorithms trained on single-reader labels are unlikely to transcend the performance of that single reader. This limitation becomes especially apparent in situations with high interobserver variability, for instance with ^18^F-PSMA-1007, where frequent unspecific bone uptake can challenge consistent labeling (5).

As unreliable labels introduce substantial uncertainty into the development and evaluation of AI algorithms, we advocate for the adoption of new standards for lesion labeling in nuclear medicine. We propose that each scan should be labeled independently by at least 2 readers, with discrepancies resolved either through consensus between the initial readers or by adjudication from one or more additional readers. Depending on the algorithm’s intended task, developers should consider integrating additional medical data, such as pathologic results, clinical information, or supporting or follow-up imaging. If only a subset of the dataset contains such enriched labels, we recommend a dedicated subgroup analysis to assess algorithm performance on this subset while carefully acknowledging potential selection bias. In addition, we emphasize that transparent reporting of the labeling methodology is essential. Crucially, the labeling methodology, including but not limited to the number and expertise of readers, software used for labeling, procedures for resolving discrepancies, and any supplementary or follow-up data used, must be explicitly reported in all publications.

To conclude, building on the recommendations by Bradshaw et al. for standardizing nuclear medicine terminology and data, we argue that high-quality, transparently reported lesion labeling is a critical addition. The value and interpretability of clinical AI algorithms largely depend on the labeling quality, an aspect that is currently often underemphasized. High-quality labeling and transparent reporting of labeling methodology should therefore become a key criterion for both authors and peer reviewers, ensuring reproducible, comparable, and clinically relevant AI algorithms.

## DISCLOSURE

No potential conflict of interest relevant to this article was reported.

## Notes

**Remco J. Poelarends**

*Isala, Zwolle, The Netherlands, and University Medical Center Groningen, Groningen, The Netherlands E-mail: r.j.poelarends@isala.nl*

**Jorn A. van Dalen**

*Isala, Zwolle, The Netherlands*

**Riemer H.J.A. Slart**

*University Medical Center Groningen, Groningen, The Netherlands*

**Brian N. Vendel**

*Isala, Zwolle, The Netherlands*

**Walter Noordzij**

*University Medical Center Groningen, Groningen, The Netherlands*

**Joris D. van Dijk**

*Isala, Zwolle, The Netherlands*
